# National Association of Dental Examiners

**Published:** 1895-06

**Authors:** Charles A. Meeker

**Affiliations:** 29 Fulton Street, Newark, N. J.


### NOTICES.

### National Association of Dental Examiners.

It is earnestly requested of the presiding officers or secretaries of the examining boards throughout the states and territories that they kindly forward to the national secretary the full list of officers, with their respective addresses.

In view of the large meeting expected at Asbury Park this coming August, the secretary desires to give every board due notice in ample time, and likewise obtain a corrected list of the officers to date.

CHARLES A. MEEKER, D.D.S., *Secretary*,
29 Fulton Street, Newark, N. J.

